# Intraoperative Identification of a Non-recurrent Laryngeal Nerve Associated With an Aberrant Right Subclavian Artery During Thyroidectomy: A Case Report

**DOI:** 10.7759/cureus.89908

**Published:** 2025-08-12

**Authors:** Hugo Pais Moreira, Victor Viegas, Hugo Louro, Susana Graça, Antónia Póvoa, Carlos Soares

**Affiliations:** 1 General Surgery, Unidade Local de Saúde Gaia/Espinho, Porto, PRT

**Keywords:** aberrant right subclavian artery, anatomical variation, arteria lusoria, dysphagia lusoria, intraoperative neuromonitoring, neck surgery, non-recurrent laryngeal nerve, recurrent laryngeal nerve injury, thyroidectomy, vagus nerve

## Abstract

The purpose of this case report is to highlight the clinical relevance of identifying a non-recurrent laryngeal nerve (NRLN), a rare anatomical variant of the inferior laryngeal nerve (ILN), in the context of thyroid surgery. The NRLN is most commonly associated with an aberrant right subclavian artery (arteria lusoria), and its presence significantly increases the risk of nerve injury during cervical procedures due to its atypical course and unexpected location.

We report the case of a 48-year-old female patient undergoing total thyroidectomy for multinodular goiter. Preoperative evaluation revealed, on computed tomography, an aberrant right subclavian artery with a retroesophageal course, consistent with an arteria lusoria and suggestive of an NRLN.

Intraoperatively, during careful dissection of the right thyroid lobe, the surgical team noted the absence of the recurrent laryngeal nerve (RLN) in its usual tracheoesophageal location. A nerve branch was identified arising directly from the cervical portion of the vagus nerve, coursing transversely towards the larynx, consistent with a right-sided NRLN.

Intraoperative neuromonitoring (IONM) was used to confirm the functional integrity of the identified nerve, preventing inadvertent injury. The surgery proceeded without complications, and the patient had an uneventful recovery with no vocal cord dysfunction or signs of nerve injury.

Beyond reinforcing the known association between arteria lusoria and NRLN, this case underscores the value of preoperative imaging as a predictive tool for nerve anomalies and highlights the critical role of IONM in identifying and preserving atypical nerve anatomy. Awareness of such variants and a structured intraoperative approach can significantly reduce the risk of iatrogenic nerve injury, informing best practices in thyroid and neck surgery.

## Introduction

Anatomical variations in the cervical and thoracic regions are relatively common, and understanding these variations is crucial for reducing the risk of iatrogenic injuries during surgical procedures. The inferior laryngeal nerve (ILN), a terminal branch of the vagus nerve, frequently follows a recurrent path, descending into the thorax before ascending towards the larynx. However, in rare cases, the ILN may take a non-recurrent course, a variation known as the non-recurrent laryngeal nerve (NRLN). The prevalence of the recurrent laryngeal nerve (RLN) anomaly is estimated to be 0.3-0.8% [1,2]. This variation is more commonly observed on the right side and is often associated with an aberrant right subclavian artery, also called arteria lusoria [2-4]. The arteria lusoria originates distally from the aortic arch and courses posteriorly to the esophagus, which may lead to esophageal compression and result in dysphagia lusoria [3,5].

In addition to arteria lusoria, other vascular anomalies, such as variations in subclavian artery branching patterns, have been documented and may further complicate cervical anatomy. Notably, the presence of a unilateral thyrovertebral trunk can alter the trajectory of neighboring neural structures, including the NRLN, increasing the risk of injury during surgeries such as thyroidectomy or parathyroidectomy. Jensen et al. (2025) highlighted that these vascular variations can substantially modify the course of the NRLN, emphasizing the need for heightened awareness when operating in this region [6,7].

Iatrogenic injury to the RLN, particularly in the presence of these anatomical variants, is a well-documented complication, and the risk of such injuries is increased when preoperative imaging fails to detect these anomalies. Detailed anatomical knowledge, a high index of suspicion, and the use of intraoperative neuromonitoring (IONM) are essential for ensuring the preservation of nerve function and minimizing surgical risk [8,9]. Based on the most recent meta-analysis focused on this issue, the pooled risk of injury to an NRLN is about 7%, with a risk ratio of approximately 3.8-fold higher compared to a normal RLN [6].

The aim of this report is to describe a case in which a right NRLN was identified intraoperatively, associated with arteria lusoria. This case underscores the critical importance of anatomical knowledge, particularly regarding vascular and neural variations, and the role of advanced technologies in preventing surgical complications. By sharing this case, we hope to highlight the need for greater awareness of these variations and the value of preoperative and intraoperative strategies in preserving nerve function during cervical surgeries.

## Case presentation

A 48-year-old female patient was referred for elective total thyroidectomy due to bilateral multinodular goiter, showing progressive growth over the past two years and causing mild anterior cervical compression. She was euthyroid, with no severe compressive symptoms such as dyspnea or dysphagia. Her medical history was unremarkable, with no previous neck surgery, radiation exposure, or known iodine deficiency. She reported no chronic medication use and had no family history of thyroid disease or malignancy.

On physical examination, there was a volumetric enlargement of the thyroid gland, more prominent on the right side, without palpable lymphadenopathy. No clinical signs of laryngeal dysfunction were observed, and phonation was normal. Cervical ultrasound revealed multiple bilateral nodules, with the largest located in the right lobe. No suspicious cervical lymph nodes were detected.

**Figure 1:**
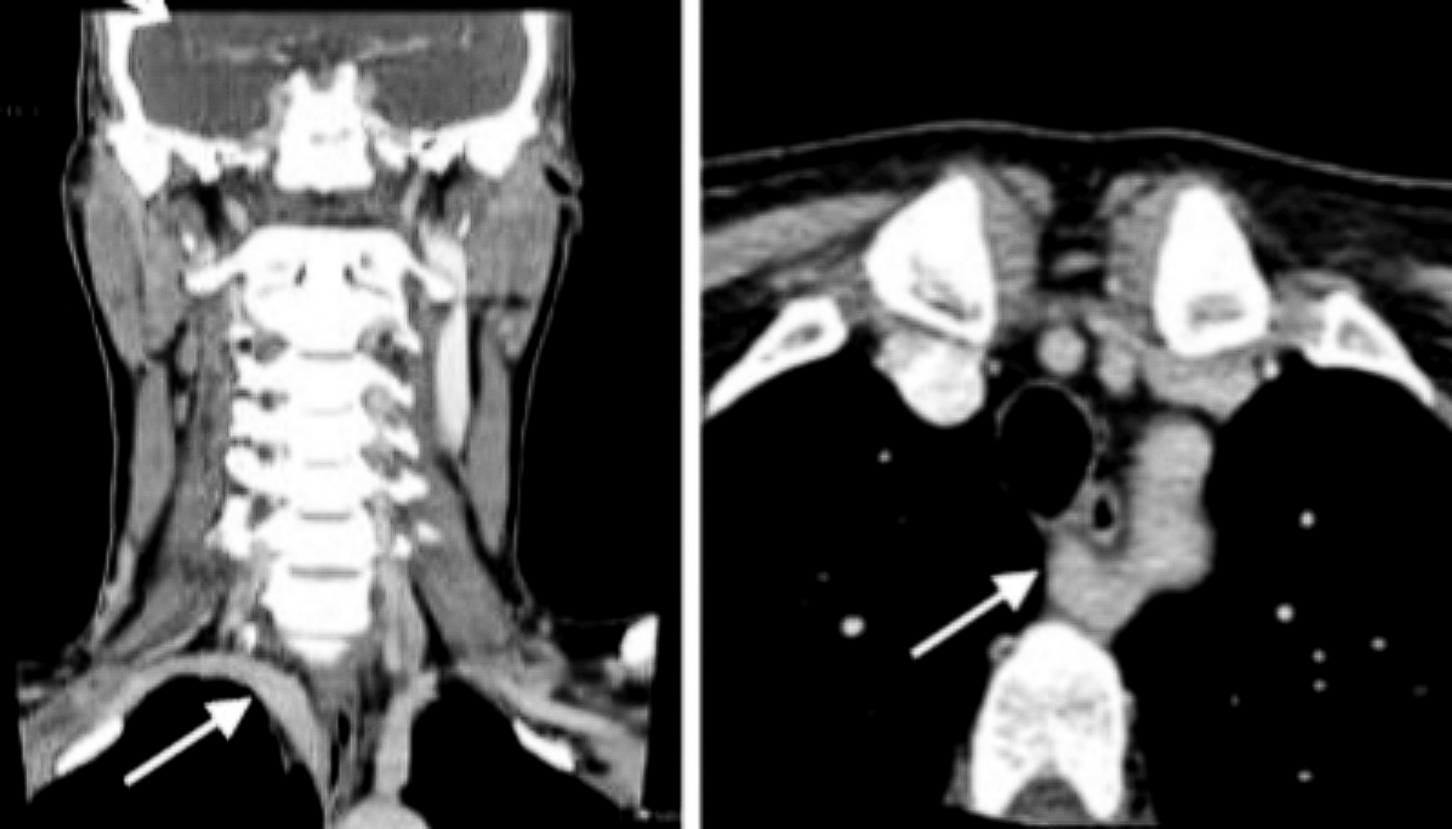
CT of arteria lusoria In the absence of the brachiocephalic trunk, the right subclavian artery emerges directly from the left side of the aortic arch and runs posteriorly to the trachea and esophagus (as indicated by the arrows).

Preoperative cervicothoracic computed tomography demonstrated an aberrant right subclavian artery with a retroesophageal course, consistent with an arteria lusoria (Figure 1).

The patient underwent a total thyroidectomy under general anesthesia with IONM. The procedure began with a standard Kocher collar incision, subplatysmal flap elevation, and division of the midline between the strap muscles to expose the thyroid gland. Dissection proceeded on the left side first, identifying and preserving the RLN and parathyroid glands.

**Figure 2:**
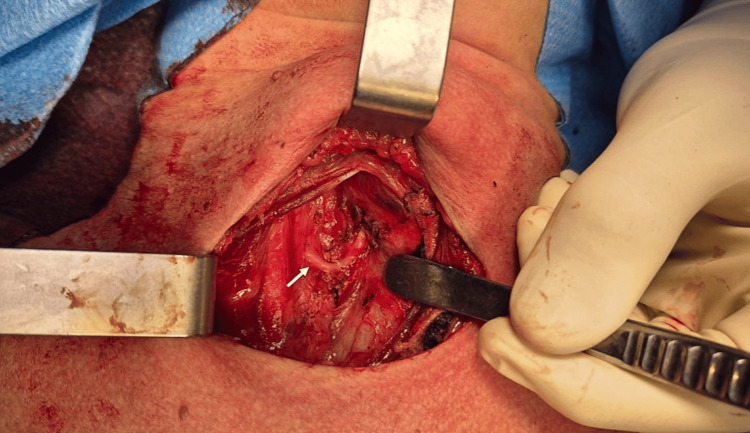
Non-recurrent laryngeal nerve After originating in the vagus nerve, it goes straight to the cricothyroid junction (as indicated by the arrow).

On approaching the right thyroid lobe, careful exploration of the tracheoesophageal groove failed to reveal the RLN in its expected location. Given the preoperative finding of an aberrant right subclavian artery, an NRLN was suspected. Meticulous dissection along the course of the vagus nerve in the cervical segment identified a nerve branch emerging directly from it, running transversely towards the larynx. Proximal stimulation of the vagus nerve with IONM confirmed a positive motor response, and the NRLN was carefully preserved throughout its course using a combination of blunt dissection and bipolar coagulation away from the nerve (Figure 2). The procedure was completed without complications.

Given the preoperative confirmation of the vascular anomaly, no additional postoperative imaging was required. The patient had an uneventful recovery, with no voice changes or signs of hypocalcemia, and was discharged on the second postoperative day.

Ethics statement

Written informed consent was obtained from the patient for publication of this case and the accompanying images. The study was conducted in accordance with institutional ethical standards and the principles of the Declaration of Helsinki.

## Discussion

**Figure 3:**
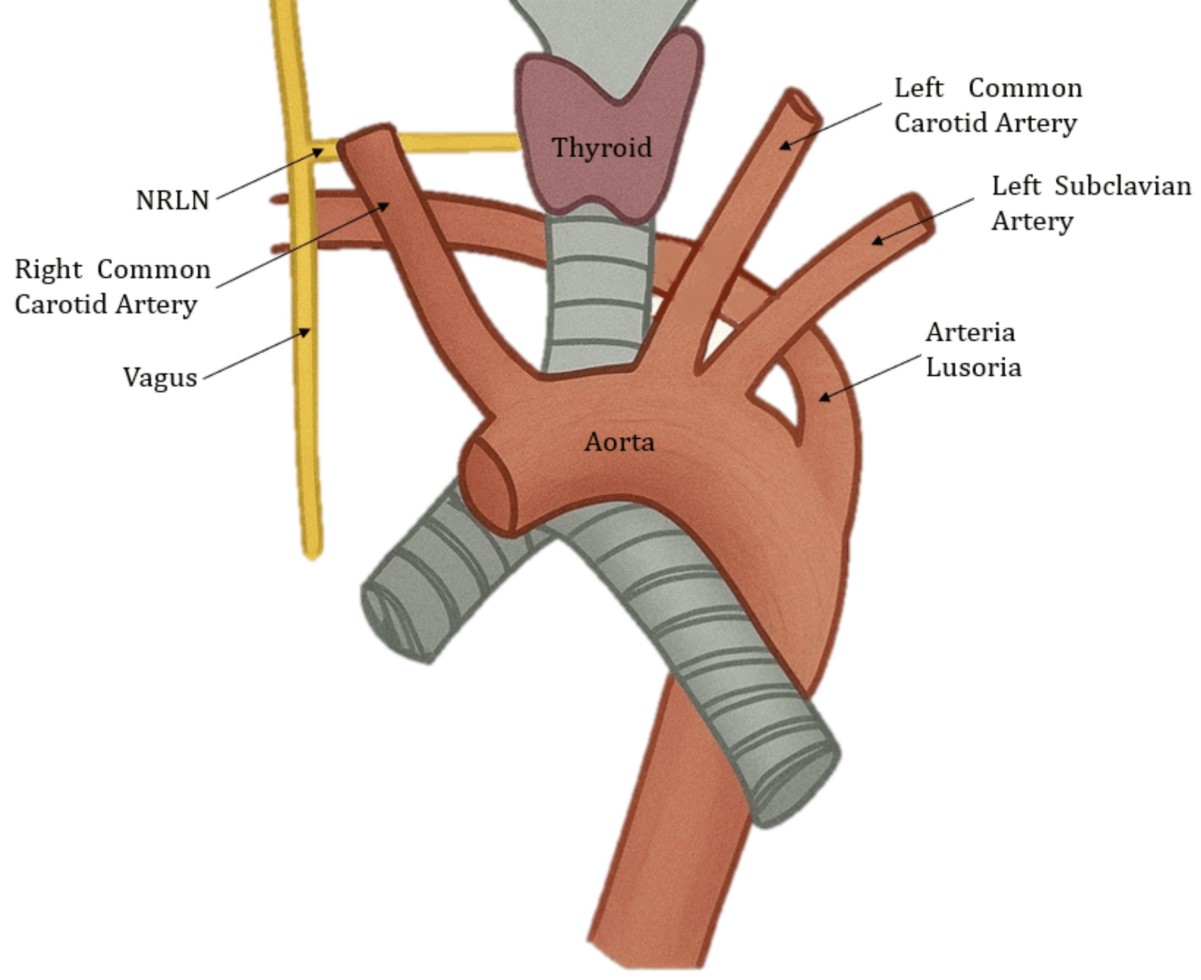
Anatomy of arteria lusoria and non-recurrent laryngeal nerve (NRLN) Illustration created by the authors. Used with permission.

The NRLN is a rare anatomical variation, with an estimated prevalence between 0.3% and 0.8% in cervical surgeries, and is almost exclusively encountered on the right side [2]. It is most commonly associated with an aberrant right subclavian artery (arteria lusoria), which originates distally from the aortic arch and follows a retroesophageal path. However, other subclavian artery branching variants, such as a right subclavian artery arising directly from the aortic arch without a retroesophageal course, have also been reported, and these can coexist with or without NRLN [4,5,8]. Such variations arise from alterations in the embryonic development of the fourth branchial arch and aortic arteries, leading to the absence of the nerve’s typical recurrent loop and a direct cervical connection from the vagus nerve to the larynx (Figure 3) [2,10].

The presence of an NRLN poses a significant challenge during thyroidectomy, as its atypical trajectory increases the risk of inadvertent injury [10]. If unrecognized, injury to the NRLN may result in vocal fold paralysis, persistent dysphonia, and considerable impairment in the patient’s quality of life [4,11,12]. Recognition of indirect preoperative signs, such as subclavian artery branching anomalies identified on imaging, can be a critical alert for the potential presence of an NRLN and may guide modifications in the surgical approach [5,8].

IIONM is a valuable adjunct for detecting such variants, especially when the nerve does not follow the conventional tracheoesophageal groove [10]. Proximal stimulation of the vagus nerve before full exposure can confirm the presence of an NRLN [8]. Nonetheless, IONM is not without limitations; it is operator-dependent, subject to false negatives due to technical issues such as electrode malposition, poor contact, or signal interference, and cannot replace meticulous anatomical dissection [13].

From a broader perspective, these considerations highlight the importance of incorporating vascular anomaly screening into preoperative imaging protocols for thyroid and parathyroid surgery. When suggestive findings such as an aberrant right subclavian artery are present, surgical planning should explicitly account for the possibility of an NRLN. Moreover, integrating these anatomical variants and their surgical implications into surgical training curricula is essential for improving recognition, decision-making, and safe dissection techniques.

In the present case, the preoperative identification of an arteria lusoria allowed the surgical team to anticipate an NRLN, adjust the dissection strategy, and employ IONM effectively, ultimately preserving nerve function [7,9]. Such a structured approach, combining targeted preoperative imaging, intraoperative vigilance, and critical knowledge of anatomical variants, can reduce complication rates and enhance functional outcomes in cervical surgery [4,9,10].

## Conclusions

The presence of an NRLN, particularly when associated with an arteria lusoria, represents a significant anatomical challenge during thyroidectomy. Preoperative recognition of vascular variants, combined with meticulous dissection and the use of intraoperative neuromonitoring, is essential to prevent nerve injury. This case highlights the value of integrating detailed preoperative imaging assessment into routine surgical planning and reinforces the need for surgical training to include recognition and management of rare anatomical variations, thereby informing future clinical decision-making and improving patient safety.
